# A Cre-Dependent CRISPR/dCas9 System for Gene Expression Regulation in Neurons

**DOI:** 10.1523/ENEURO.0188-21.2021

**Published:** 2021-08-18

**Authors:** Nancy V. N. Carullo, Jenna E. Hinds, Jasmin S. Revanna, Jennifer J. Tuscher, Allison J. Bauman, Jeremy J. Day

**Affiliations:** Department of Neurobiology and Evelyn F. McKnight Brain Institute, University of Alabama at Birmingham, Birmingham, AL 35294

**Keywords:** Cre/Lox, CRISPR/dCas9, transcription

## Abstract

Site-specific genetic and epigenetic targeting of distinct cell populations is a central goal in molecular neuroscience and is crucial to understand the gene regulatory mechanisms that underlie complex phenotypes and behaviors. While recent technological advances have enabled unprecedented control over gene expression, many of these approaches are focused on selected model organisms and/or require labor-intensive customization for different applications. The simplicity and modularity of clustered regularly interspaced short palindromic repeats (CRISPR)-based systems have transformed genome editing and expanded the gene regulatory toolbox. However, there are few available tools for cell-selective CRISPR regulation in neurons. We designed, validated, and optimized CRISPR activation (CRISPRa) and CRISPR interference (CRISPRi) systems for Cre recombinase-dependent gene regulation. Unexpectedly, CRISPRa systems based on a traditional double-floxed inverted open reading frame (DIO) strategy exhibited leaky target gene induction even without Cre. Therefore, we developed an intron-containing Cre-dependent CRISPRa system (SVI-DIO-dCas9-VPR) that alleviated leaky gene induction and outperformed the traditional DIO system at endogenous genes in HEK293T cells and rat primary neuron cultures. Using gene-specific CRISPR sgRNAs, we demonstrate that SVI-DIO-dCas9-VPR can activate numerous rat or human genes (*GRM2*, *Tent5b*, *Fos*, *Sstr2*, and *Gadd45b*) in a Cre-specific manner. To illustrate the versatility of this tool, we created a parallel CRISPRi construct that successfully inhibited expression from a luciferase reporter in HEK293T cells only in the presence of Cre. These results provide a robust framework for Cre-dependent CRISPR-dCas9 approaches across different model systems, and enable cell-specific targeting when combined with common Cre driver lines or Cre delivery via viral vectors.

## Significance Statement

This manuscript reports a novel set of clustered regularly interspaced short palindromic repeats (CRISPR) tools for Cre-dependent transcriptional targeting in neurons and non-neuronal dividing cells. Our results demonstrate that these tools perform well at multiple gene targets and can be used for both transcriptional activation or repression. Compared with traditional Cre-dependent overexpression or knock-out models, Cre-dependent CRISPR activation (CRISPRa) and CRISPR interference (CRISPRi) provide several advantages, including targeting of one or multiple endogenous genomic loci, titration of effect size, and bidirectional regulation using the same CRISPR sgRNA. Further, SVI-DIO-dCas9 tools can be used in existing transgenic animal models or wild-type models via viral delivery. This advance enables applications in less common animal models and can be used to target endogenous genomic loci for fine-tuned transcriptional activation or repression.

## Introduction

Genetic and epigenetic targeting are fundamental strategies to study gene regulation and function, and also provide novel therapeutic avenues for genetic diseases. In recent years, clustered regularly interspaced short palindromic repeats (CRISPR) systems have revolutionized the field as tools for site-specific DNA and RNA editing (Jinek et al., 2012; Cong et al., 2013; Savell and Day, 2017; Pickar-Oliver and Gersbach, 2019). In these systems, a Cas nuclease is directed to target DNA by an engineered CRISPR single guide RNA (sgRNA), resulting in nuclease-mediated cleavage of the targeted nucleic acid sequence. In addition to CRISPR-Cas9 systems, a number of new approaches have been developed based on a mutated, catalytically dead Cas9 (dCas9). CRISPR-dCas9 strategies target specific genomic loci without causing double-strand breaks, and can instead be fused to an array of different effector proteins (Savell and Day, 2017). dCas9 systems are commonly used to induce transcriptional activation (CRISPRa) or interference (CRISPRi), epigenetic modifications, chromatin looping, tagging, or can be used as an anchor system to deliver non-coding RNAs (CRISPR-Display; Hilton et al., 2015; Konermann et al., 2015; Shechner et al., 2015; Yeo et al., 2018; Chen et al., 2019; Savell et al., 2019a; Carullo et al., 2020).

DNA recombinases are commonly used to enable inversion, deletion, or integration of transgenes in a cell-specific manner. The Cre/Lox system is a commonly used recombination approach in which the Cre recombinase recognizes specific 34-bp palindromic Lox sites within a DNA sequence (Anton and Graham, 1995; Gibb et al., 2010). Cre-mediated inversion or deletion occurs when flanking Lox sites are oriented in opposing or parallel orientations, respectively. Importantly, Cre recombinase expression driven under cell type-specific promoters enables targeted gene manipulation of these cell populations (Madisen et al., 2012; Daigle et al., 2018). Due in part to the ease and versatility of this system, hundreds of different Cre-driver transgenic lines have been generated for cell-targeted approaches (Gong et al., 2007; Madisen et al., 2012; Daigle et al., 2018). Likewise, Cre-dependent genetic FLEX switches (flip-excision; also known as double-inverted open reading frame, or DIO switches) invert DNA sequences to enable gene activation or silencing (Schnütgen et al., 2003). This approach has widely been used in neuroscience, enabling cell type-specific optogenetic and chemogenetic activation, overexpression or knock-down of transgenes, and perturbation of endogenous genes (Kühn et al., 1995; Tsien et al., 1996; Gong et al., 2007; Atasoy et al., 2008; Madisen et al., 2012; Erwin et al., 2020).

While CRISPR/dCas9 approaches have enabled targeted induction of transcriptional or epigenetic states at selected genes, inducible cell type-specific CRISPR tools based on these platforms remain limited (Kumar et al., 2018; Bäck et al., 2019). For example, most currently available transgenic CRISPR-dCas9 lines are limited to the mouse (Zhou et al., 2018; Gemberling et al., 2021), and existing rat transgenic lines rely on Cre-dependent CRISPR guide RNA constructs to achieve cell type specificity (Bäck et al., 2019). These approaches are limited to the specific transgenic model system, and require costly crosses with Cre lines and time-consuming animal colony management. Similar approaches based on viral delivery of CRISPR machinery have so far been limited to constitutive transgene expression (Zheng et al., 2018; Savell et al., 2019a), or only enable genome editing via the introduction of double-strand breaks (Kumar et al., 2018). Here, we developed novel Cre-dependent CRISPRa and CRISPRi systems that incorporate a synthetic intron into the dCas9 transgene. Creating an intron-containing dCas9 construct offers two advantages: (1) introns can increase transgene expression compared with intron-lacking counterparts (Shaul, 2017); and (2) introns provide a non-coding locus for insertion sites of Lox sequences without disturbing the integrity of the coding protein. By separating the dCas9 transgene into two segments with a short SV40 intron (SVI), we created CRISPRa and CRISPRi systems in which the first segment of dCas9 is double-floxed and inverted. Importantly, this SV40 intron is only 97 bp, and therefore does not substantially affect construct size or compatibility with viral vector delivery. This approach prevents leaky expression of functional CRISPR constructs by requiring Cre-induced inversion to orient both dCas9 segments into an open reading frame. Furthermore, the construct is designed for easy rearrangement and replacement of promoters and effector fusions and can be applied for various CRISPR/dCas9 systems.

## Materials and Methods

### Cultured neuron experiments

Primary rat neuronal cultures were generated from embryonic day 18 rat striatal tissue as described previously (Savell et al., 2019a). Briefly, cell culture wells were coated overnight at 37°C with poly-L-lysine (0.05 mg/ml for culture wells supplemented with up to 0.05 mg/ml laminin) and rinsed with diH_2_O. Dissected tissues were incubated with papain for 25 min at 37°C. After rinsing in HBSS, a single-cell suspension of the tissue was re-suspended in Neurobasal media (Invitrogen) by trituration through a series of large to small fire-polished Pasteur pipets. Primary neuronal cells were passed through a 100 μm cell strainer, spun and re-suspended in fresh media. Cells were then counted and plated to a density of 125,000 cells per well on 24-well culture plate with or without glass coverslips (60,000 cells/cm). Cells were grown in Neurobasal media plus B-27 and L-glutamine supplement (complete neurobasal media) for 11 days in vitro (DIV) in a humidified CO_2_ (5%) incubator at 37°C.

For virus experiments, cells were transduced with lentiviruses on DIV4 or DIV5. All viruses had a minimum titer of 1 × 10^9^ GC/ml, with a target multiplicity of infection (MOIs) of at least 1000. After an 8- to 16-h incubation period, virus-containing media was replaced with conditioned media to minimize toxicity. A regular half-media change followed on DIV8. On DIV11, transduced cells were imaged and virus expression was verified before RNA extraction. EGFP and mCherry expression was also used to visualize successful transduction using a Nikon TiS inverted epifluorescence microscope.

### RNA extraction and RT-qPCR

Total RNA was extracted (RNAeasy kit, QIAGEN) with DNase treatment (RNase free DNase, QIAGEN), and reverse-transcribed (iScript cDNA Synthesis kit, Bio-Rad). cDNA was subject to qPCR for genes of interest, as described previously (Savell et al., 2016). A list of PCR primer sequences is provided in Table 1.

**Table 1:** Sequences of primers and sgRNAs

cDNA primers (RT-qPCR)					
Gene	Species	Forward sequence	Reverse sequence	Template length	Notes
*Gapdh* mRNA	*H. Sapiens*	TGTCAAGCTCATTTCCTGGTAT	CTCTCTTCCTCTTGTGCTCTTG	133 bp	Intron- spanning
*GRM2* mRNA	*H. Sapiens*	CATTCCTGCCCATCTTCTATGT	GTGGCTAACCACGTTCTTCT	161 bp	Intron- spanning
*Gapdh* mRNA	*R. norvegicus*	ACCTTTGATGCTGGGGCTGGC	GGGCTGAGTTGGGATGGGGACT	217 bp	Intron- spanning
*Tent5b* mRNA	*R. norvegicus*	CCAGCAGATTGTACAGGTAGT	CTCACTCTGCAGGTCCATCT	163 bp	Intron- spanning
*Gadd45b* mRNA	*R. norvegicus*	GACAACGCGGTTCAGAAGAT	TCCTCCTCTTCTTCGTCTATGG	173 bp	Intron- spanning
*Sstr2* mRNA	*R. norvegicus*	CGTGGAAAAGCAAGATGTCAC	TATGGCTCTGTCTGGTTGGA	213 bp	Intron- spanning
gDNA primers					
Gene	Location	Forward sequence	Reverse sequence	Template length	
Human *GAPDH*	642 bp upstream of *GAPDH* TSS	GAGCCTCGAGGAGAAGTTCC	GGACCCTTACACGCTTGGAT	167 bp	
Rat *Gapdh*	174 bp upstream of *Gapdh* TSS	GGTCGGAGCCCACACGCTTG	TCCCGCTCGGCTCATCCAGT	161 bp	
“P1” inverted DIO-dCas9	Forward primer in beginning of dCas9 with reverse primer in WRPE	GGCCGATGCTGTACTTCTTG	TGTAATCCAGAGGTTGATTGTC	324 bp	
“P2” correct orientation, recombined DIO-dCas9	Forward primer in hSYN with reverse primer in beginnig of dCas9	GAGTCGTGTCGTGCC	GGCCGATGCTGTACTTCTTG	180 bp (constitutive), 242-bp DIO	
“P3” inverted SVI-DIO	Forward primer in hSYN with reverse primer in inverted first segment of dCas9	GAGTCGTGTCGTGCC	ATTCTGCGGCGGCAGGAAGA	359 bp	
“P4” recombined SVI-DIO	Intron-spanning in recombined SVI-DIO	ACCTTCCGCATCCCCTACTA	TCGAAGTTGGTCATCCGCTCG	262 bp (spliced)/ 421 bp (unspliced)	
CRISPR guide RNA (sgRNA)					
Genomic locus	Species	Target sequence	Genomic coordinates		
*lacZ*	*E. coli*	TGCGAATACGCCCACGCGAT	*E. coli* strain 99–3165:3,886, 546–3,886,565		
*Tent5b*	*R. norvegicus*	GAATTCGGTCCGAAGAGAAG	Rn6: Chr5: 155,354,031		
*Gadd45b*	*R. norvegicus*	GGCGGTGGAGCAGCTGCTGG	Rn6: Chr7: 11,647,911–11,647,930		
*Sstr2*	*R. norvegicus*	TGCGCCGCTAGCATTGGCCG	Rn6: Chr10: 102,136,282–102,136,263		
*Fos*	*R. norvegicus*	GAGCGGAACAGAGAAACTGG	Rn6: Chr6: 109,300,373–109,300,354		
*Fos*	*R. norvegicus*	GTGAAAGTTACAGACTGAGA	Rn6: Chr6: 109,300,259–109,300,278		
*Fos*	*R. norvegicus*	GTGCAGCGCAAGGGGGGAGC	Rn6: Chr6: 109,300,064–109,300,045		
*Fos*	*R. norvegicus*	GCCTAATGTTGCACTGATTT	Rn6: Chr6: 109,299,775–109,299,794		

### CRISPR-dCas9 construct design

To achieve transcriptional activation or inactivation, lentivirus-compatible plasmids were engineered to express dCas9 fused to either VPR or KRAB-MeCP2, based on existing published plasmids [Addgene plasmid #114196 (Savell et al., 2019a); Addgene plasmid #155365 (Duke et al., 2020)]. A Cre-dependent DIO version of the dCas9-VPR construct was generated by insertion of LoxP and Lox2272 sequences flanking the dCas9-VPR cassette. dCas9-VPR was PCR-amplified to insert additional restriction sites (KpnI, BmtI, and BspDI) to allow for LoxP and Lox2272 insertion and was subsequently inserted in reverse orientation to create an intermediate construct via sequential digest and ligation (AgeI, EcoRI). LoxP and Lox2272 sites were amplified from a DIO construct (Addgene plasmid #113685; Don et al., 2017) to create restriction sites (KpnI and BmtI around one set; BspDI and KpnI around another set) and were inserted into the intermediate construct via sequential restriction digest and ligation (KpnI, BmtI, BspDI, and EcoRI). Intron-containing dCas9-VPR was constructed by the insertion of a gBlock containing the SV40 intron into the original dCas9-VPR plasmid via Gibson assembly (Gibson Assembly kit, New England BioLabs). The SVI-FLEX construct was built via Gibson assembly of the original dCas9-VPR backbone and two gBlocks encoding the SV40 intron sequence, LoxP and Lox2272 sites (sequences based on DIO construct described above) and dCas9-part1 in inverted orientation. The intron-containing CRISPRi construct (SVI-dCas9-KRAB-MeCP2) was built via restriction digest and Gibson assembly (SfiI, EcoRI, and Xhol, Gibson Assembly kit, New England BioLabs) of the KRAB-MeCP2 and SVI-dCas9-VPR constructs. A Cre-encoding construct (Addgene plasmid #49056; Kaspar et al., 2002) was used to amplify and insert a Cre transgene into a lentivirus compatible backbone that contained the hSYN promoter and expressed mCherry for visualization via Gibson assembly. To create an additional Cre construct, mCherry was replaced with GFP via sequential digest and ligation (EcoRI and XhoI). dCas9-VPR-expressing constructs were co-transduced with sgRNA-containing constructs. Gene-specific sgRNAs were designed using an online sgRNA tool, provided by the Zhang Lab at MIT (crispr.mit.edu) and inserted in a previously described lentivirus compatible sgRNA scaffold construct (Addgene plasmid #114199; Savell et al., 2019b). To ensure specificity, all CRISPR crRNA sequences were analyzed with the National Center for Biotechnology Information’s (NCBI) Basic Local Alignment Search Tool (BLAST) and Cas-OFFinder (http://www.rgenome.net/cas-offinder/). sgRNAs were designed to target *GRM2*, *Tent5b*, *Sstr2*, *Gadd45b*, and *Fos*, respectively. A list of the target sequences is provided in Table 1. crRNA sequences were annealed and ligated into the sgRNA scaffold using the BbsI or BsmBI cut site. Plasmids were sequence-verified with Sanger sequencing; final crRNA insertion was verified using PCR. Lentivirus-compatible SYN-SVI-DIO-dCas9-VPR and SYN-SVI-DIO-dCas9-KRAB-MeCP2 plasmids are available on Addgene (plasmids #164576 and #170378).

### Lentivirus production

Viruses were produced in a sterile environment subject to BSL-2 safety by transfecting HEK293T cells with specified CRISPR-dCas9 plasmids, the psPAX2 packaging plasmid, and the pCMV-VSV-G envelope plasmid (Addgene plasmids #12260 and #8454) with FuGene HD (Promega) for 40–48 h as previously described (Savell et al., 2019b). Viruses were purified using filter (0.45 μm) and ultracentrifugation (25,000 rpm, 1 h 45 min) steps. Viral titer was determined using a qPCR Lentivirus Titration kit (Lenti-X, qRT-PCR Titration kit, Takara). For smaller scale virus preparation, each sgRNA plasmid was transfected in a 12-well culture plate as described above. After 40–48 h, lentiviruses were concentrated with Lenti-X concentrator (Takara), resuspended in sterile PBS, and used immediately. Viruses were stored in sterile PBS at −80°C in single-use aliquots.

### HEK293T cell culturing and transfection

HEK293T cells were obtained from American type Culture Collection (ATCC; catalog #CRL-3216, RRID:CVCL_0063) and cultured in standard HEK media: DMEM (DMEM high glucose, pyruvate; Invitrogen 11995081) supplemented with 10% bovine serum (Qualified US Origin; BioFluid 200–500-Q) and 1 U penicillin-streptomycin (Invitrogen 15140122). Cells were maintained in T75 or T225 tissue flasks. At each passage, cells were trypsinized for 1–3 min (0.25% trypsin and 1 mm EDTA in PBS, pH 7.4) at room temperature. For transfection experiment cells were plated in 24-well plates and transfected with FuGene HD (Promega).

### Luciferase assay

Bidirectional regulation by SVI-DIO CRISPRa and CRISPRi machinery was examined using a previously described *Fos* luciferase reporter plasmid (Duke et al., 2020). A total of 80,000 HEK293T cells were plated in 500 μl HEK Media. After cells reached 40–50% confluence, 500 ng total plasmid DNA was transfected with 1.5 μl FuGene HD (Promega) as follows: 50 ng of luciferase plasmid, 450 ng in 1:2 molar ratio of total sgRNA:CRISPRa or CRISPRi plasmid. A luciferase glow assay was performed according to manufacturer’s instructions 40 h following transfection (Thermo Scientific Pierce Firefly Glow assay; Thermo Scientific 16177). Cells were lysed in 100 μl 1× luciferase cell lysis buffer while shaking at low speed and protected from light for 30 min; 20 μl of cell lysate was then added to an opaque 96-well microplate (Corning 353296) and combined with 50 μl 1× D-luciferin working solution supplemented with 1× firefly signal enhancer (Thermo Scientific Pierce Firefly Signal Enhancer; Thermo Scientific 16180). Following a 10-min dark incubation period to allow for signal stabilization, luminescence was recorded using a Synergy 2 Multi-Detection Microplate Reader (BioTek). Luminescence in dCas9-KRAB-MeCP2 and dCas9-VPR experiments was recorded with a read height of 1 mm, 1-s integration time, and 135- or 100-ms delay, respectively. Representative images of luciferase reporter activity assays were captured using an Azure c600 imager (Azure Biosystems).

## Results

### Creation and validation of a traditional FLEX-CRISPRa system

**Figure 1.**
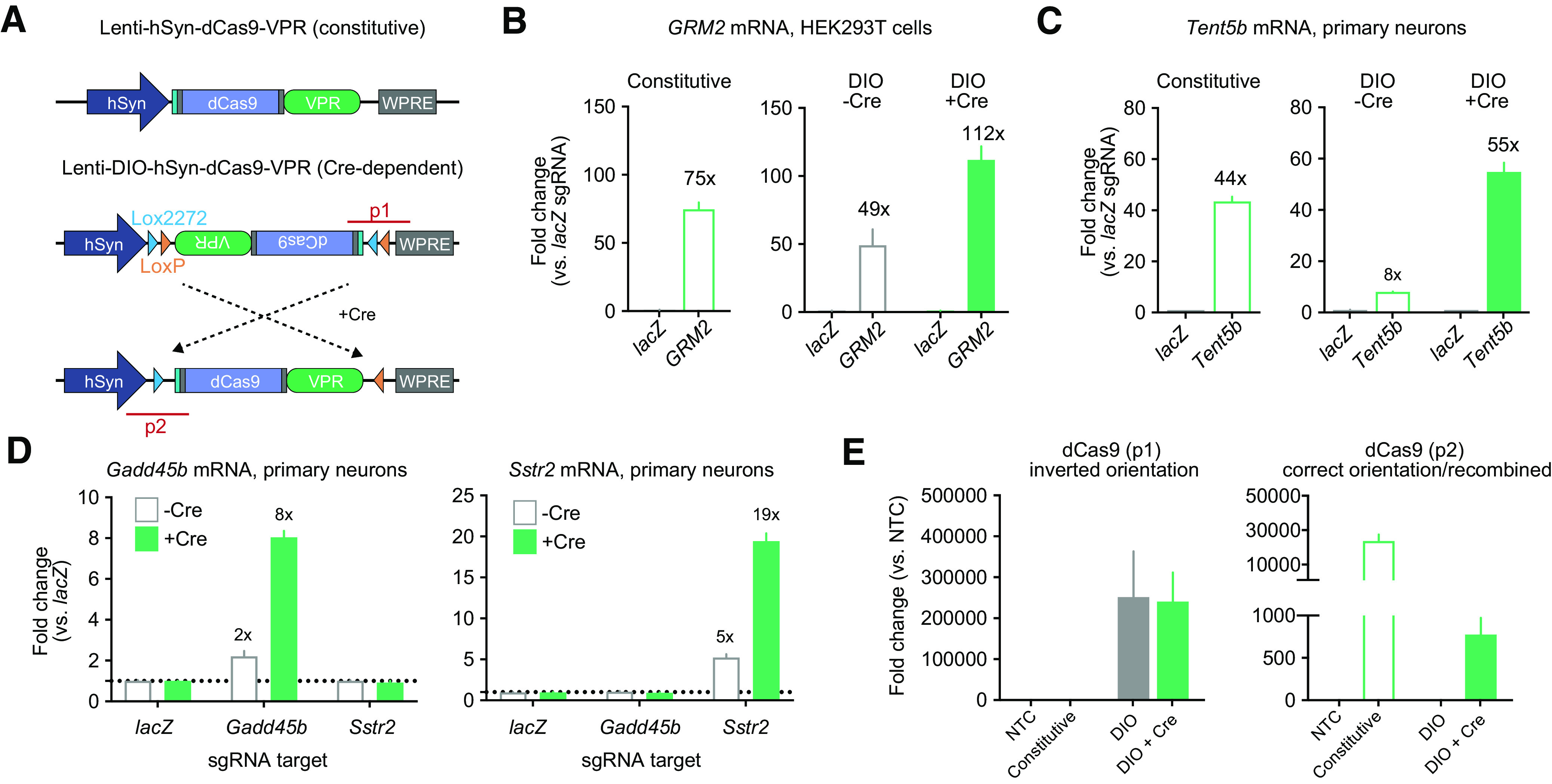
Design and validation of a DIO-dCas9-VPR CRISPRa plasmid. ***A***, Illustration of CRISPRa construct designs for a traditional constitutive dCas9-VPR (top) and a Cre-dependent double-floxed inverted open reading frame (DIO) dCas9-VPR (bottom). PCR products for primer sets used in panel e are illustrated as p1 and p2 (red). ***B***, In transfected HEK293T cells, the constitutive dCas9-VPR construct induced the endogenous target gene *GRM2* by 75-fold compared with the non-targeting *lacZ* sgRNA control (Welch’s *t* test *t*_(2)_ = 14.43*, p *=* *0.0048). DIO-dCas9-VPR significantly induced transcription of *GRM2* with and without expression of the Cre construct (*n *=* *3 per group, two-way ANOVA *F*_(1,8)_ = 32.89, *p *=* *0.0004). ***C***, Lentiviral expression of constitutive dCas9-VPR construct as well as the DIO-dCas9-VPR and Cre constructs in rat striatal neurons revealed increased mRNA for the target gene *Tent5b* (Welch’s *t* test for constitutive *t*_(2)_ = 22.81, *p *=* *0.0019, two-way ANOVA for DIO with *n *=* *3 per group, *F*_(1,8)_ = 487.9, *p *<* *0.0001). ***D***, Additional CRISPRa target genes tested in neurons demonstrated leaky induction in the absence of Cre recombinase (two-way ANOVA with *n *=* *3 per group for *Gadd45b F*_(1,12)_ = 91.03, *p *<* *0.0001, and for *Sstr2 F*_(1,12)_ = 142.1, *p *<* *0.0001). ***E***, qPCR on transfected HEK293T cell DNA showed high levels of the inverted dCas9-VPR cassette with and without lentiviral Cre expression (p1, left, *n *=* *4 per group, Kruskal–Wallis test *F*_(3,12)_ = 13.06 *p *<* *0.0001). Inversion into the correct orientation only occurred in the presence of Cre (p2, right, *n *=* *4 per group, Kruskal–Wallis test *F*_(3,12)_ = 12.11 *p *=* *0.0002). Constitutive VPR and DIO groups (with and without Cre) are compared with a non-transduced control (NTC). Data expressed as mean ± SEM.

AAV-driven FLEX systems are among the most common Cre/Lox approaches used in neurons, enabling inducible expression by combination with Cre-driver animal models. While this strategy has proven to be a versatile approach for cell-specific targeting, few tools have been described for Cre-dependent CRISPR manipulation. Given the flexibility of CRISPRa and CRISPRi systems for transcriptional manipulation at endogenous loci, development of Cre-dependent platforms for these systems are of interest. To create a Cre-inducible system for transcriptional activation, we adapted a CRISPRa tool recently validated in neurons (Savell et al., 2019a) using a common DIO strategy. In this DIO system, an inverted dCas9-VPR cassette was flanked by LoxP and Lox2272 sites (Fig. 1*A*), enabling Cre-dependent inversion of dCas9-VPR into the correct orientation followed by the excision of one antiparallel Lox site to prevent further inversion events. To validate this system in dividing cells, HEK293T cells were co-transfected with either a constitutively active dCas9-VPR construct or the DIO-dCas9-VPR construct, in tandem with sgRNA plasmids targeting either the human *GRM2* promoter, or a non-targeting control (sgRNA for the bacterial *lacZ* gene). The DIO-dCas9-VPR construct was tested with or without transfection of a Cre-2A-mCherry plasmid that was driven under the human synapsin (hSYN) promoter (Fig. 1*B*). Constitutive activation of the endogenous *GRM2* gene yielded a 75-fold increase of *GRM2* mRNA compared with a non-targeting *lacZ* control sgRNA. While the Cre-dependent DIO-dCas9-VPR system was able to induce *GRM2* mRNA transcription by 112-fold, we also observed a 49-fold increase in the absence of Cre. Next, to examine gene induction in neurons, we transduced rat primary striatal neuron cultures with lentiviruses expressing sgRNAs, CRISPR machinery, and Cre recombinase (Fig. 1*C*). CRISPR sgRNAs targeting the *Tent5b* (also known as *Fam46b*) promoter resulted in strong upregulation of *Tent5b* mRNA with both the constitutive and Cre-dependent constructs. However, in neurons the DIO system also exhibited leaky expression, indicated by an 8-fold increase of *Tent5b* mRNA in the absence of Cre. *Tent5b* is a highly inducible gene which could be more susceptible to baseline/leaky induction compared with other genes. Therefore, we tested the DIO-dCas9-VPR system at two other genes including a gene encoding the neuropeptide receptor *Sstr2* and DNA-damage inducible gene *Gadd45b* (Fig. 1*D*). Although these genes are less inducible than *Tent5b* (with induction rates of 8- to 19-fold compared with *lacZ* controls), we still observed non-specific gene induction in the absence of Cre (2- to 5-fold induction without Cre). While we chose efficient gRNAs validated in prior studies (Savell et al., 2019a, 2020; Carullo et al., 2020), it is possible that the differences in inducibility across gene targets are driven by the respective gRNA properties rather than inherent inducibility of the target genes. Alternately, this difference may correspond to variation in gene inducibility, driven by baseline expression, gene length, or other features of transcript regulation. Nevertheless, the induction of target genes in the absence of Cre recombinase is likely because of leaky expression of the DIO-dCas9-VPR transgene, since no transgene inversion could be detected in PCR verification using genomic DNA isolated from cells lacking Cre (Fig. 1*E*). These results are consistent with recent evidence that DIO transgenes can be expressed in the inverted orientation at low levels in the absence of Cre recombinase (Fischer et al., 2019).

### Intron insertion into dCas9 increases expression and enables creation of a split-dCas9 FLEX system

**Figure 2.**
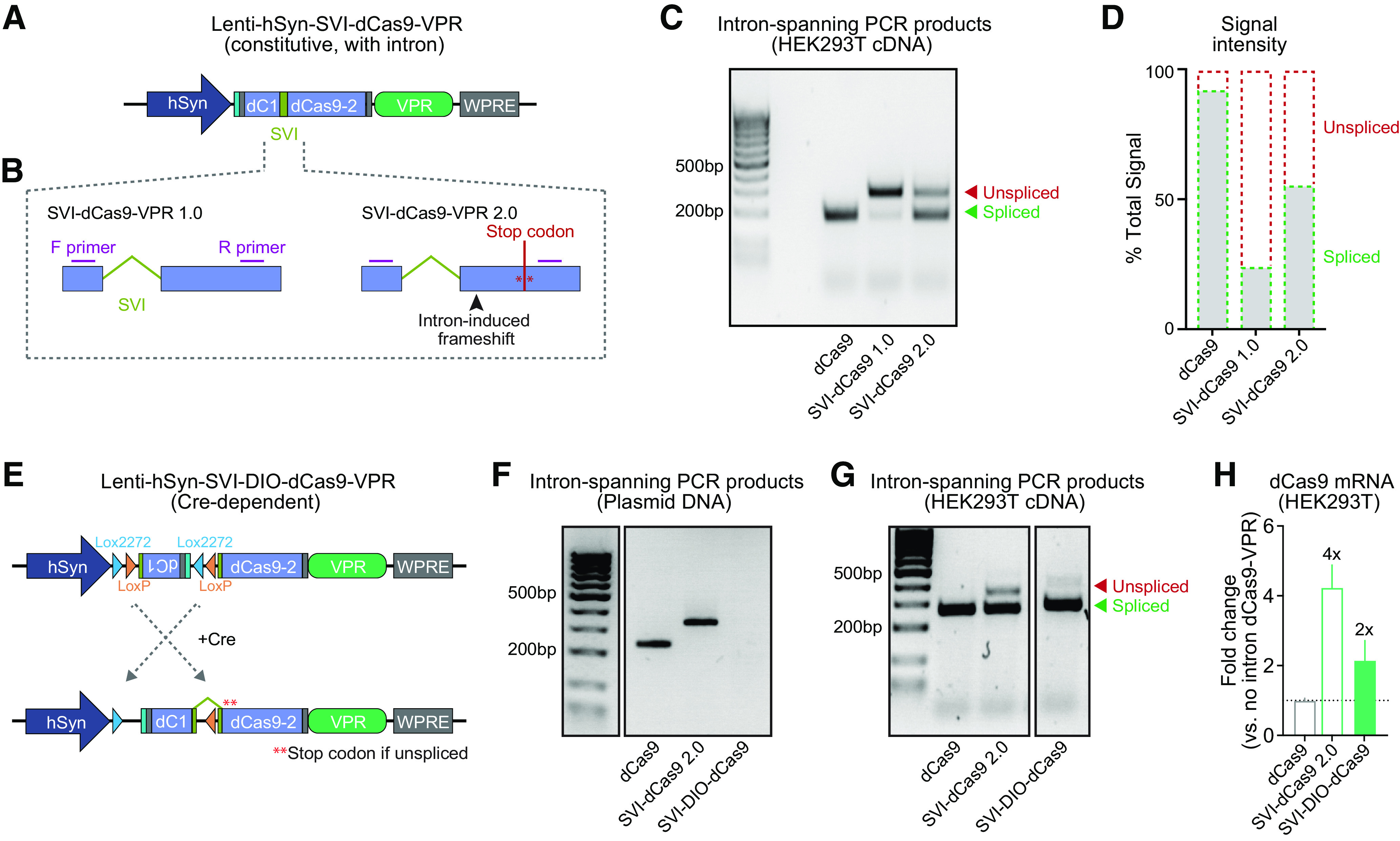
Design of an intron-containing, Cre-dependent CRISPRa system. ***A***, Illustration of CRISPR construct design for an intron-containing constitutive dCas9-VPR cassette. ***B***, Design of the two different intron positions. ***C***, SVI-dCas9-VPR 1.0 and 2.0 intron positions were tested in HEK293T cells. PCR amplification of cDNA with primers spanning the intron within dCas9 showed more efficient splicing of the SVI-dCas9-VPR 2.0 construct. ***D***, Quantification of PCR product intensities shown in panel ***C***. ***E***, Illustration of a Cre-dependent SVI-DIO-dCas9-VPR construct in which the first dCas9 segment is double-floxed and inverted. ***F***, PCR amplification of plasmid DNA with intron-spanning primers verify that no product results from the SVI-DIO-dCas9-VPR plasmid before recombination. ***G***, PCR amplification of cDNA following HEK293T cell transfection with dCas9-VPR, SVI-dCas9-VPR, and SVI-DIO-dCas9-VPR with Cre showed that SVI-DIO-dCas9-VPR transfected cells express spliced dCas9-VPR transcripts in the presence of Cre recombinase. ***H***, Intron insertion increases dCas9-VPR mRNA expression, detected with RT-qPCR for a common region within dCas9 (*n *=* *8 per group). Kruskal–Wallis test *F*_(3,24)_ = 14.62, *p *=* *0.0007; Multiple comparisons tests *p *=* *0.0003 for SVI-dCas9 2.0 versus constitutive dCas9, and *p *=* *0.0357 for SVI-DIO-dCas9 (with Cre recombinase) versus constitutive dCas9. Data expressed as mean ± SEM.

Given that an inverted dCas9-VPR cassette still resulted in target gene induction in the absence of Cre, we next aimed to design a system that would prohibit expression of the full length dCas9-VPR fusion protein without Cre-mediated recombination. To achieve this goal, we designed an intron-containing FLEX system. First, we inserted a small SV40 intron (SVI) into the dCas9 sequence to create a constitutively active, intron-containing construct (SVI-dCas9-VPR) in which the dCas9 cassette is divided into two segments by the SV40 intron (Fig. 2*A*). We created two versions (1.0 and 2.0) of this SVI-dCas9-VPR construct to determine optimal intron positioning and maximal splicing efficiency. While the intron was only slightly shifted between versions 1.0 and 2.0, SVI-dCas9-VPR 2.0 created a frameshift immediately after the intron, resulting in a premature stop codon within <100 bases if the product was not spliced (Fig. 2*B*). PCR amplification from cDNA with primers spanning this intron sequence following HEK293T plasmid transfection revealed that both constructs produced detectable dCas9 expression. However, splicing efficiency of the SVI-dCas9-VPR 2.0 version was higher as compared with SVI-dCas9-VPR 1.0 (Fig. 2*C*,*D*). This variability is not surprising given the known effects of intron placement on splicing efficiency (Shaul, 2017). While we still detected low levels of unspliced mRNA using the SVI-dCas9-VPR 2.0 construct, this is potentially driven by nascent RNA that has not yet undergone splicing rather that intron retention (which would result in a premature stop codon).

Using the more efficient SVI-dCas9-VPR 2.0, we next generated a split-dCas9 DIO cassette by inverting and flanking the first segment of dCas9 with LoxP and Lox2272 sites to prevent leaky transgene expression (Fig. 2*E*). With only part of the dCas9 cassette inverted, Cre-dependent inversion and recombination is required to yield a full-length functional dCas9-VPR fusion protein. Using the same intron-spanning primer set as in previous validations, we compared PCR products amplified from the original constitutive dCas9-VPR, the intron-containing constitutive VPR (SVI-dCas9-VPR 2.0), and the new Cre-dependent SVI-dCas9-VPR (SVI-DIO-dCas9-VPR) plasmids. As predicted, the SVI-dCas9-VPR 2.0 plasmid yielded a longer product compared with the original dCas9-VPR with no intron (Fig. 2*F*), because of the 97 bp intron in the SVI-dCas9-VPR 2.0 plasmid. However, because the SVI-DIO-dCas9-VPR contains an inverted dCas9 segment, the validation primers target the same strand and yield no PCR product. These results further validate the absence of non-specific recombination following plasmid transfection.

We next transfected HEK293T cells with each of these three constructs to compare recombination, expression, and splicing efficiency of the SVI-DIO-dCas9-VPR construct. The SVI-DIO-dCas9-VPR groups also received a Cre-EGFP plasmid that was driven under the hSYN promoter to initiate recombination. PCR amplification of cDNA generated from the transfected HEK293T experiments with intron-spanning primers revealed strong signals for the short, spliced PCR product for all three groups (Fig. 2*G*). This indicates that all three constructs were expressed in HEK293T cells and that both SVI-dCas9-VPR 2.0 and SVI-DIO-dCas9-VPR transgenes generated mRNA transcripts with efficient splicing and intron exclusion. We further observed that, in HEK293T cells, SVI-dCas9-VPR and SVI-DIO-dCas9-VPR mRNA was expressed at levels 4-fold and 2-fold higher (respectively) than observed with the constitutive dCas9-VPR plasmid (Fig. 2*H*). Together, these experiments demonstrate that this newly developed intron-containing FLEX system is capable of efficient splicing and transcription and that Cre-dependent recombination is required for inversion and expression of the dCas9-VPR transgene.

### Intron-containing FLEX system drives Cre-dependent CRISPR targeting with no leaky gene induction

**Figure 3.**
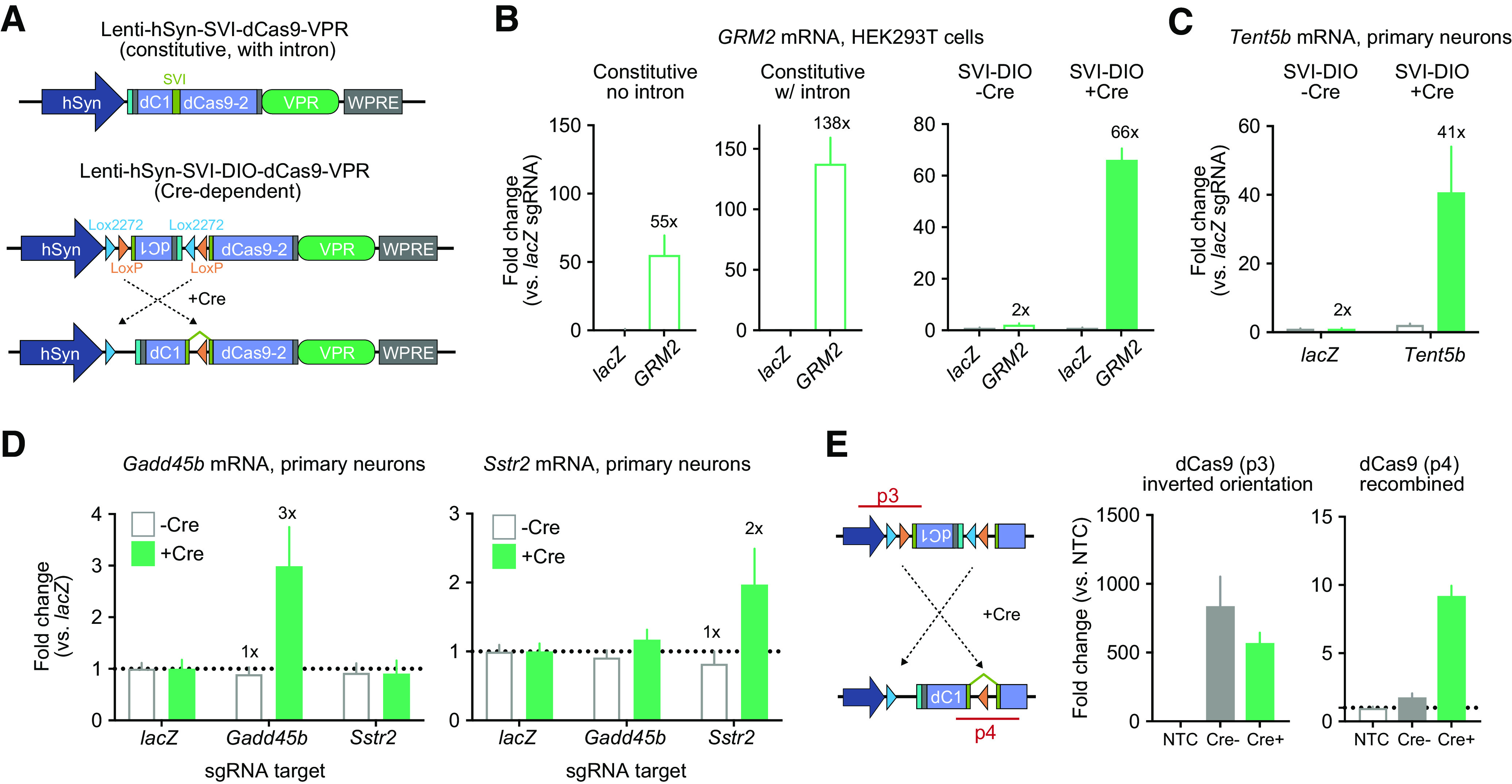
Validation of the SVI-DIO-dCas9-VPR CRISPRa system. ***A***, Illustration of CRISPR construct designs for the intron-containing SVI-dCas9-VPR (top) and a Cre-dependent double-floxed inverted open reading frame (DIO) SVI-dCas9-VPR (bottom). ***B***, In transfected HEK293T cells the constitutive VPR construct with and without the SV40 intron induced the endogenous target gene *GRM2* by 55-fold and 138-fold compared with the non-targeting *lacZ* control (Welch’s *t* test for constitutive *t*_(2)_ = 3.846, *p *=* *0.0614, and constitutive with intron *t*_(2)_ = 6.322, *p *=* *0.0241). SVI-DIO-dCas9-VPR significantly induced transcription of *GRM2* with but not without the Cre construct (*n *=* *3 per group, two-way ANOVA *F*_(1,8)_ = 179.5, *p *<* *0.0001). ***C***, Lentiviral expression of SVI-DIO-dCas9-VPR and Cre in striatal neurons increased mRNA for the target gene *Tent5b* (*n *=* *10 per group, two-way ANOVA *F*_(1,36)_ = 34.97, *p *<* *0.0001). ***D***, Additional target genes tested in neurons demonstrated specific induction only in presence of Cre recombinase (two-way ANOVA with *n *=* *3 per group for *Gadd45b F*_(1,48)_ = 4.578, *p *=* *0.0375, and for *Sstr2 F*_(1,48)_ = 6.615, *p *=* *0.0133). ***E***, Illustration of primer and PCR product position for recombination validation experiments (left). qPCR on transduced neuronal DNA revealed high levels of the inverted dCas9-VPR cassette (p3, middle, *n *=* *2 per group, Kruskal–Wallis test *F*_(2,4)_ = 3.714 *p *=* *0.2) with and without Cre. Inversion into the correct orientation only occurred in the presence of Cre (p4, right, *n *=* *2 per group, Kruskal–Wallis test *F*_(2,4)_ = 4.57 *p *=* *0.0667). SVI-DIO-dCas9-VPR groups with and without Cre are compared with a non-transduced control (NTC). Data expressed as mean ± SEM.

Next, we sought to test this newly developed intron-containing FLEX CRISPR system in vitro to validate Cre-specific expression and gene induction (Fig. 3*A*). Using a similar experimental design as in the DIO-dCas9-VPR experiments (Fig. 1), we transfected HEK293T cells with plasmids expressing constitutive dCas9-VPR, SVI-dCas9-VPR, and SVI-DIO-dCas9-VPR constructs, along with sgRNA targeting the GRM2 gene promoter (or *lacZ* control). Both the intron-containing SVI-dCas9-VPR and constitutive dCas9-VPR caused strong induction of GRM2 compared with their respective *lacZ* controls (Fig. 3*B*). Notably, unlike the classic DIO-dCas9-VPR construct, the SVI-DIO-dCas9-VPR plasmid exhibited very little induction without Cre (2-fold) and strong induction with Cre (66-fold).

Similarly, targeting the highly inducible *Tent5b* gene in cultured striatal neurons using lentiviral transgene delivery resulted in strong upregulation of *Tent5b* mRNA with the Cre-dependent constructs (41-fold), but not in the absence of Cre (2-fold; Fig. 3*C*). These patterns of Cre-dependent induction without leaky effects were also observed for both of the moderately inducible target genes tested in Figure 1 (*Sstr2* and *Gadd45b*; Fig. 3*D*). PCR amplification of genomic DNA that was extracted from transduced primary neurons further validated that recombination required expression of Cre (Fig. 3*E*). Together, these data demonstrate that this novel SVI-DIO-dCas9-VPR construct mitigates the leaky gene induction seen in conventional DIO systems for CRISPRa approaches while maintaining the capacity for robust and specific gene induction.

### Cre-dependent CRISPRa and CRISPRi constructs modulate expression from a luciferase reporter

**Figure 4.**
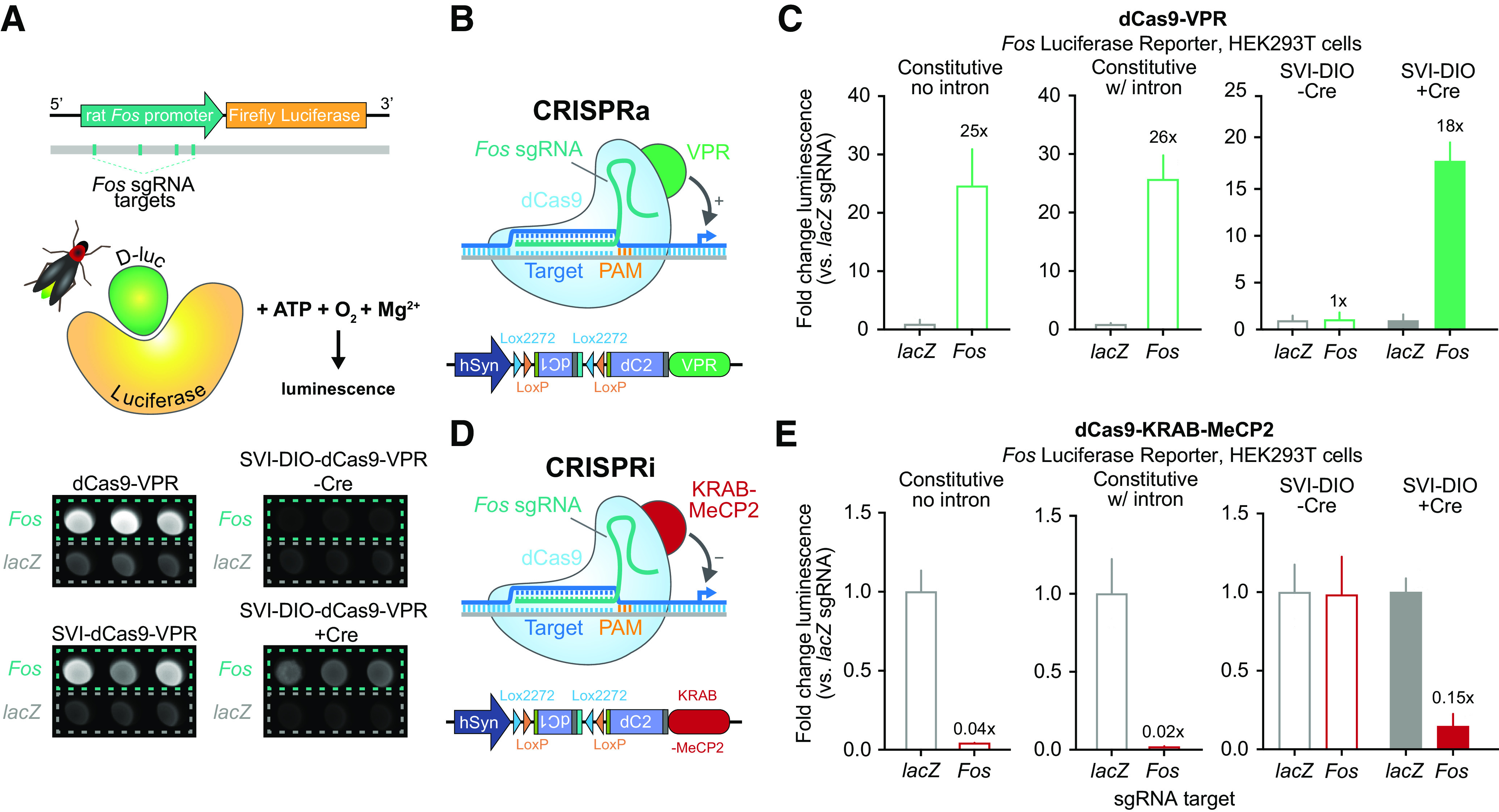
Validation of SVI-DIO-dCas9-VPR CRISPRa and SVI-DIO-dCas9-KRAB-MeCP2 CRISPRi systems using a *Fos* luciferase reporter. ***A***, top, Illustration of *Fos* Firefly Luciferase plasmid with respective *Fos* sgRNA targets. Bottom, raw luminescence data from CRISPRa luciferase assay illustrates increased luminescence after targeting the *Fos* promoter with constitutive (left) and Cre-inducible dCas9-VPR fusions. SVI-DIO-dCas9-VPR only increases luciferase activity in the presence of Cre recombinase. ***B***, Illustration of SVI-DIO-dCas9-VPR CRISPRa construct used for Fos Firefly Luciferase targeting. ***C***, In transfected HEK293T cells, constitutive dCas9-VPR constructs with and without an SV40 intron induced a significant increase in luminescence after targeting *Fos* compared with the non-targeting *lacZ* control (Welch’s *t* test for constitutive VPR no intron *t*_(2)_ = 11.38, *p *<* *0.0001, and constitutive with intron *t*_(2)_ = 18.53, *p *<* *0.0001). SVI-DIO-dCas9-VPR significantly increased luminescence only in the presence of Cre (*n *=* *9 per group, two-way ANOVA *F*_(1,32)_ = 535.4, *p *<* *0.0001). ***D***, CRISPRi (KRAB-MeCP2) construct coupled with *Fos* Firefly Luciferase plasmid. ***E***, After transfection of HEK293T cells, the constitutive KRAB-MeCP2 construct with and without the SV40 intron decreased luminescence when paired with *Fos* sgRNAs (Welch’s *t* test for constitutive KRAB-MeCP2 no intron *t*_(2)_ = 12.29, *p *=* *0.0065, and constitutive with intron *t*_(2)_ = 10.78, *p *=* *0.0001). SVI-DIO-dCas9-KRAB-MeCP2 decreased luminescence only in the presence of Cre (*n *=* *6 per group, two-way ANOVA *F*_(1,20)_ = 40.37, *p *<* *0.0001). Data expressed as mean ± SEM.

To further validate functionality of our SVI-DIO-dCas9-VPR construct, we tested this system at a luciferase reporter plasmid driven by the rat *Fos* promoter (Fig. 4*A*,*B*; Duke et al., 2020). Luciferase assays are a common luminescence reporter system used to validate effects of an effector molecule on gene expression. Luciferase, when paired with its substrate D-luciferin and in the presence of ATP, O_2_, and Mg^2+^, produces bioluminescence that can provide insight into the direct transcriptional activity at a regulatory element driving luciferase expression. HEK293T cells were co-transfected with plasmids expressing the *Fos* luciferase plasmid, constitutive or Cre-inducible CRISPRa plasmids, and plasmids expressing sgRNAs targeting either the rat *Fos* promoter or a non-targeting *lacZ* control. Additionally, the SVI-DIO-dCas9-VPR construct was transfected with a Cre-EGFP plasmid. Notably, the constitutive dCas9-VPR constructs with and without the SV40 intron increased luminescence 26-fold and 25-fold change, respectively, when compared with *lacZ*. Likewise, the SVI-DIO-dCas9-VPR increased luminescence in a similar range (18-fold), but only in the presence of Cre recombinase (Fig. 4*C*). Consistent with our previous results in neurons and HEK293T cells (Fig. 3), this Cre-dependent CRISPRa construct exhibited no leaky induction in the absence of Cre, confirming the utility of this approach for Cre-specific expression and gene induction.

While gene overexpression is useful for gain-of-function experiments, CRISPR/dCas9 approaches benefit from the ability to use the same sgRNA for both gene activation and repression, based on the identity of the protein fused to dCas9. However, given the possibility of DIO transgene expression in reverse orientation (Fischer et al., 2019), leaky expression is likely not limited to dCas9-VPR based systems but could also occur in other CRISPR-DIO based systems. In order to generate Cre-dependent system for CRISPRi, we created a parallel Cre-dependent construct in which dCas9 is fused to a KRAB-MeCP2 repressor domain that results in robust gene silencing when targeted to gene promoters (Yeo et al., 2018; Duke et al., 2020; Fig. 4*D*). Following similar transfection and luciferase assay protocols used for our CRISPRa system, we found that constitutive KRAB-MeCP2 both with and without the SV40 intron effectively inhibited luminescence from the *Fos*-luciferase reporter in HEK293T cells (Fig. 4*E*). Likewise, the SVI-DIO-dCas9-KRAB-MeCP2 inhibited luciferase activity only in the presence of Cre recombinase. Together, these results demonstrate the capacity of our SVI-DIO CRISPR constructs to target and modulate gene expression at specific transcriptional regulatory regions in a Cre-dependent manner. Likewise, because luciferase reporter assays require successful translation of luciferase protein, these experiments also demonstrate the capability of this system to bidirectionally regulate protein levels in addition to mRNA of targeted genes. Furthermore, adaptation of this SVI-DIO system for multiple CRISPR approaches, such as CRISPRi, highlights the potential and versatility of this tool for cell type-specific and gene-specific regulation in future experiments.

## Discussion

The mammalian brain consists of heterogeneous cell populations with distinct characteristics and functions. Technologies to study gene function in specific tissues, brain regions, or cell populations are therefore necessary to understand their role in behavior and disease. For example, cell type-selective promoters have been used for specific targeting of excitatory neurons (*Camk2a*; Mima et al., 2001), astrocytes (*Gfap*; Morelli et al., 1999), and cell populations that express specific enzymes or receptors such as tyrosine hydroxylase (*Th*; Savitt et al., 2005), dopamine or serotonin transporters (*Slc6a3*, *Slc6a4*; Zhuang et al., 2005; Bäck et al., 2019), or dopamine receptors (*Drd1*, *Drd2*; Lobo et al., 2006; Zhang et al., 2006). In this study we developed and optimized Cre-dependent CRISPRa and CRISPRi systems that enable gene and cell type-specific transcriptional activation and inactivation, respectively. In cultured dividing and non-diving cell lines, we demonstrate that this SVI-DIO-dCas9 based CRISPRa system can activate highly inducible endogenous genes in HEK293T cells (*GRM2* gene) and striatal neurons (*Tent5b*), without undesired gene induction in the absence of Cre recombinase (Figs. 3, 4). This system also performed well when targeted to moderately inducible genes such as *Sstr2* and *Gadd45b* in neurons (Fig. 3), demonstrating a wide range of effect size and application possibilities. Furthermore, successful activation and repression of a *Fos* luciferase reporter in HEK293T cells using SVI-DIO CRISPR constructs demonstrates the versatility of this machinery to provide robust bidirectional control over gene expression and protein levels.

Using a classic DIO strategy in which the entire dCas9-VPR cassette was double-floxed and inverted, we observed leaky target gene induction in the absence of Cre (Fig. 1). While it is unclear what drives this leaky expression, it is conceivable and in line with recent findings that DIO transgenes can be expressed in the inverted orientation in the absence of Cre (Fischer et al., 2019). To avoid this leaky expression, we generated a novel SVI-DIO-dCas9 system in which dCas9 was broken up into two segments by an SV40 intron. This intron provided a location for insertion of Lox sites to invert and double-flox the first dCas9 fragment while leaving the second fragment undisturbed. Without Cre-mediated recombination, the dCas9 segments remain in opposing orientations and are thus unable to yield functional dCas9 protein. Consistent with observations that introns can facilitate nuclear mRNA export and translation processes and potentially increase functional protein levels (Shaul, 2017), we found that intron insertion increased dCas9-VPR mRNA expression following plasmid transfection (Fig. 2*H*). Critically, because of the small size of this intron (97 bp), it is unlikely to substantially limit viral packaging and could easily be applied to increase transgene expression in other constructs.

One of the first cell type-specific CRISPR systems used in neurons was based on Cre-dependent expression of CRISPR sgRNAs. Bäck et al., developed a Lox-stop-Lox based sgRNA construct and used it for CRISPR-Cas9 mediated gene knock-out (Bäck et al., 2019). This study tested this approach in transgenic rats that expressed Cre recombinase under the rat dopamine transporter (*Slc6a3*) promoter to specifically target and knock-out the *Th* gene in dopaminergic neurons. While this approach allowed for cell type-specific expression of sgRNA constructs, a concern with similar systems is the untargeted overexpression of Cas9 or dCas9 fusion proteins, which could potentially cause unintended and non-specific effects on gene expression.

Our system extends this previous work in two ways. First, the SVI-DIO-dCas9 approach alleviates some of these concerns, as the dCas9 construct itself is Cre dependent, and therefore, functional fusion proteins are not expressed without Cre recombinase. Second, our SVI-DIO-dCas9 system is compatible with traditional validated sgRNA constructs, which can be multiplexed for effect size titration at a single gene (Savell et al., 2019a). Additionally, this approach is also compatible with more complex sgRNA arrays that target entire gene programs (Savell et al., 2020). Thus, this study adds another approach for gene regulation to the CRISPR toolbox, while also outlining a novel intron/DIO strategy to avoid leaky Cre-independent transgene expression. Compared with traditional Cre-dependent overexpression vectors, Cre-dependent CRISPR strategies provides several advantages as they enable targeting of one or multiple endogenous genomic loci and titration of effect size for more physiological expression levels.

Prior work has incorporated temporal specificity into Cre/Lox systems, either via use of optically or chemically inducible proteins. For example, selective estrogen receptor modulator (SERM) inducible systems have been generated by fusion of the ligand binding domain of the ER to Cre (Cre-ER). In this approach, a mutated version of the mouse ER that binds tamoxifen but not estrogen was used to create a Tamoxifen-inducible Cre system (Danielian et al., 1993; Metzger and Chambon, 2001). Activity of Cre recombinase can therefore be activated or inactivated on Tamoxifen injections, increasing temporal control of gene editing events.

In addition to cell type-specificity, selective promoters can be used to drive expression in response to stimulation or experience. Bacterial artificial chromosome (BAC) transgenic animals were generated to express GFP or channelrhodopsin (ChR2) under an immediate early gene promoter such as *Fos* or *Arc* (Guenthner et al., 2013). Using activity-induced approaches in combination with our SVI-DIO-dCas9 system would allow for experience-dependent genetic and epigenetic manipulations.

Cre/Lox systems are also among the most common approaches for intersectional circuit-specific manipulations in neuroscience. For example, in recent work, circuit-specific CRISPR genome editing was achieved by injection of a Cre-dependent Cas9 viral vector in the nucleus accumbens (NAc) and injection of a monosynaptic rabies virus expressing a sgRNA targeting the *Fosb* gene in the ventral hippocampus (vHPC; Eagle et al., 2020). While the sgRNA virus successfully spread through all hippocampal projections, significant knock-out of *Fosb* occurred only in vHPC neurons that projected to the NAc. In future studies, our SVI-DIO-dCas9 system could similarly be used to gain a better understanding of projections between specific cell types in the brain. The intron-containing effector construct could be delivered to one brain region and a Cre-expressing construct to the second brain region through a monosynaptic rabies virus or retrograde AAV serotype. Genetic or epigenetic editing would then only occur in cells with projections between the two targeted brain regions without leaky expression or side effects related to effector protein overexpression in non-targeted cells.

While the use of our SVI-DIO-dCas9 system in Cre driver animal models is an expected application, this approach does not require transgenic organisms. In addition to sgRNA and SVI-DIO-dCas9-VPR or SVI-dCas9-KRAB-MeCP2 delivery, a separate construct expressing Cre can be delivered as necessary to target brain tissues. Additionally, this intron-containing dCas9 provides a basic framework that can be customized and combined with a number of effector proteins to introduce a variety of genetic or epigenetic modifications. The system can easily be customized for expression in various cell types and brain regions and adjusted for inducible efforts and enhanced temporal specificity.

## Synthesis

Reviewing Editor: Michael Michaelides, NIDA-NIH Decisions are customarily a result of the Reviewing Editor and the peer reviewers coming together and discussing their recommendations until a consensus is reached. When revisions are invited, a fact-based synthesis statement explaining their decision and outlining what is needed to prepare a revision will be listed below. The following reviewer(s) agreed to reveal their identity: Jonathan Ploski.

Reviewer 1

The manuscript is very well written and is technologically sound. I only have a few minor comments.

1. The authors mention that genes are, “highly inducible”. As written, it is unclear if the authors intend to mean that there is something inherent to the gene that makes them more or less inducible via CRISPRa. If instead they meant that in their hands CRISPRa was capable of inducing the genes more or less, they might want to clarify this.

Of note: Since the authors only examined 1 gRNA per gene, it’s possible that the observed inducibility is a product of better or worse functioning gRNAs. Had they examined other gRNAs, they might have gotten different results.

2. Figure 2C - why do you think that intron splicing isn’t 100%? It would be valuable to the reader to address this in the discussion.

Reviewer 2

Please see comments/edits in annotated PDF forwarded to editors via email.
